# Supplementary material for: Cellular development and evolution of the mammalian cerebellum
Source: Nature. 2023 Nov 29;625(7996):788–96. doi: 10.1038/s41586-023-06884-x (PMC10808058; doi:10.1038/s41586-023-06884-x)
Supplement: Supplementary file 1 — This file contains Supplementary Methods and additional references. [file 41586_2023_6884_MOESM1_ESM.pdf]

nature portfolio

https://doi.org/10.1038/s41586-023-06884-x

Supplementary information

Cellular development and evolution of the mammalian cerebellum

In the format provided by the authors and unedited

SUPPLEMENTARY INFORMATION

Cellular development and evolution of the mammalian cerebellum

Mari Sepp[1,15]*, Kevin Leiss[1,15]*, Florent Murat[1,2], Konstantin Okonechnikov[3,4], Piyush Joshi[3,4,5], Evgeny Leushkin[1], Lisa Spänig[3,4,5], Noe Mbengue[1], Céline Schneider[1], Julia Schmidt[1], Nils Trost[1], Maria Schauer[6], Philipp Khaitovich[7], Steven Lisgo[8], Miklós Palkovits[9], Peter Giere[6], Lena M. Kutscher[3,5], Simon Anders[1,10], Margarida Cardoso-Moreira[11], Ioannis Sarropoulos[1,14,16]*, Stefan M. Pfister[3,4,12,13,16]* & Henrik Kaessmann[1,16]*

[1]Center for Molecular Biology of Heidelberg University (ZMBH), DKFZ-ZMBH Alliance, Heidelberg, Germany.

[2]INRAE, LPGP, Rennes, France.

[3]Hopp-Children's Cancer Center Heidelberg (KiTZ), Heidelberg, Germany.

[4]Division of Pediatric Neurooncology, German Cancer Research Center (DKFZ) and German Cancer Consortium (DKTK), Heidelberg, Germany.

[5]Developmental Origins of Pediatric Cancer Junior Group, German Cancer Research Center (DKFZ) and German Cancer Consortium (DKTK), Heidelberg, Germany.

[6]Museum für Naturkunde Berlin, Leibniz Institute for Evolution and Biodiversity Science, Berlin, Germany.

[7]NHC Key Laboratory of Diagnosis and Treatment on Brain Functional Diseases, The First Affiliated Hospital of Chongqing Medical University, Chongqing, China.

[8]Biosciences Institute, Newcastle University, Newcastle, UK.

[9]Human Brain Tissue Bank, Semmelweis University, Budapest, Hungary.

[10]BioQuant, Heidelberg University, Heidelberg, Germany.

[11]Evolutionary Developmental Biology Laboratory, Francis Crick Institute, London NW1 1AT, UK.

[12]Department of Pediatric Hematology and Oncology, Heidelberg University Hospital, Heidelberg, Germany.

[13]National Center for Tumor Diseases (NCT) Heidelberg, Germany.

[14]Present address: Wellcome Sanger Institute, Cambridge, UK.

[15]These authors contributed equally to this work.

[16]These authors jointly supervised this work.

*e-mail: m.sepp@zmbh.uni-heidelberg.de, k.leiss@zmbh.uni-heidelberg.de, i.sarropoulos@zmbh.uni-heidelberg.de, s.pfister@kitz-heidelberg.de, h.kaessmann@zmbh.uni-heidelberg.de

TABLE OF CONTENTS

METHODS 3
Data reporting. 3
Sample collection and ethics statement. 3
Dissections. 4
Preparation of nuclei. 5
RNA extraction and sample quality control. 6
Library preparation and sequencing. 6
Genome annotations. 7
General data processing and quality control. 7
Data integration and clustering. 8
Cell type annotation and label transfer. 9
External sc/snRNA-seq datasets 12
Quantification of cell type abundances and ratios 13
Bayes modelling of cell type abundance differences 15
Identification of overdispersed genes. 16
Pseudoages. 16
Stage correspondences across species. 17
Histology. 18
Multiplexed single molecule *in situ* hybridisation data generation and analyses. 19
Immunohistochemistry. 20
Gene expression scores. 23
Cell type lineage assignments for ventricular zone neuroblasts. 24
Integration of data by subsets. 25
Cross-species correlations and comparisons to the adult mouse data. 25
Principal components analysis. 26
Marker gene identification. 27
Pseudotemporal ordering. 27
Gene expression trajectories. 28
Presence/absence expression differences. 29
Gene expression cell type-specificity. 33
Gene ontology and pathway enrichment analyses. 33
Inference of transcription factor regulon activities 34
Adult bulk RNA-sequencing data analyses. 35
Essential and disease-associated genes. 35
General statistics and plots. 36
SUPPLEMENTARY REFERENCES 36

METHODS

Data reporting.

No statistical methods were used to predetermine sample size. At least 2 biological replicates were generated for each developmental stage (except human 20 wpc). All samples are listed in Supplementary Table 1, and an overview of the samples is given in Extended Data Fig. 1a. Human sample size was based on the number of individuals available, and comparable sample size was used for mouse and opossum. The experiments were not randomized and investigators were not blinded to allocation during experiments and outcome assessment.

Sample collection and ethics statement.

The human prenatal samples used in this study were donated voluntarily to the MRC Wellcome Trust Human Developmental Biology Resource (HDBR; UK) by women who had an elective abortion and had given written informed consent to donate fetal tissues for research. The prenatal samples had normal karyotypes and were classified as belonging to a particular Carnegie stage or week post conception (wpc) according to their external physical appearance and measurements. The human postnatal samples are from the University of Maryland Brain and Tissue Bank of National Institutes of Health NeuroBioBank (USA), Chinese Brain Bank Center (CBBC) in Wuhan and Lenhossék Human Brain Program, Human Brain Tissue Bank at Semmelweis University (Hungary). Informed consent for the use of tissues for research was obtained in writing from donors or their family. All postnatal samples come from healthy non-affected individuals defined as normal controls by the corresponding brain bank. The use of human samples was approved by an ERC Ethics Screening panel (associated with H.K.'s ERC Consolidator Grant 615253, OntoTransEvol) and ethics committees in Heidelberg (authorization S-220/2017), North East-Newcastle & North Tyneside (REC reference 18/NE/0290), London-Fulham (REC reference 18/LO/0822), Ministry of Health of Hungary (No.6008/8/2002/ETT) and Semmelweis University (No.32/1992/TUKEB).

RjOrl:SWISS time-mated pregnant mice (*Mus musculus*), litters at postnatal days 0-14 and adult mice were purchased from Janvier Labs (France). Bl6N mice, used only in immunohistochemistry experiments, were purchased from the Jackson Laboratory (USA) and bred at the DKFZ Central Animal Laboratory, Heidelberg. Gray short-tailed opossums (*Monodelphis domestica*) were bred in a colony in Museum für Naturkunde Berlin, Leibniz Institute for Evolution and Biodiversity Science (Germany) or Texas Biomedical Research Institute (USA; Supplementary Table 1). The animals were housed under a 12h/12h dark/light cycle (reversed for opossums) in a temperature (20-24 °C mouse; 24-26 °C opossum) and humidity (40-65% mouse, 60-65% opossum) controlled room with *ad libitum* access to food and water. Mouse and opossum stages were dated according to the time of copulation (E, embryonic day) or birth (P, postnatal day). The adult mice were sacrificed by cervical dislocation, the pups by decapitation. Opossums were sacrificed by isoflurane overdose. All animal procedures were performed in compliance with national and international ethical guidelines for the care and use of laboratory animals, and were approved by the local animal welfare authorities: Heidelberg University Interfaculty Biomedical Research Facility (T-63/16, T-64/17, T-37/18, T-23/19), DKFZ Central Animal Laboratory (DKFZ383), Vaud Cantonal Veterinary Office (No.2734.0) and Berlin State Office of Health and Social Affairs, LAGeSo (T0198/13, ZH104).

Dissections.

Mouse and opossum cerebella were dissected as whole or in 2 halves as described[25]. Mouse E10.5 and E11.5 and opossum E14.5 cerebella were pooled from 2-3 littermates. (Supplementary Table 1). For human 7-11 wpc samples whole cerebellar primordia were collected and divided into halves or representative fragments (ca 25% of the primordium cut perpendicular to its long axis; Supplementary Table 1). For human 17-20 wpc samples, fragments of the cerebellum were collected. Newborn, infant and toddler samples include fragments of the cerebellar cortex. Adult human samples were dissected from the posterior lobe of the cerebellar hemispheres (crus I and II), vermis (lobules VI-VIII), flocculonodular lobe and dentate nucleus using the micropunch technique[56]. Developmental samples were kept in ice-cold PBS during the dissection, most of the

meninges were removed and the samples were snap-frozen in liquid nitrogen and stored at -80 °C. If available, samples from both sexes were used for data production.

Preparation of nuclei.

The nuclei were extracted as described[57] with modifications. Briefly, the frozen tissue was homogenized on ice in 250 mM sucrose, 25 mM KCl, 5 mM $MgCl_2$, 10 mM Tris-HCl (pH 8), 0.1% IGEPAL, 1 µM DTT, 0.4 U/µl Murine RNase Inhibitor (New England BioLabs), 0.2 U/µl SUPERase-In (Thermo Fisher Scientific), cOmplete Protease Inhibitor Cocktail (Roche) by trituration and/or using a micropestle. After 5 minutes of incubation the remaining bits of unlysed tissue were pelleted by centrifugation at 100g for 1 minute at 4°C. The cleared homogenate was centrifuged at 400g for 4 minutes to pellet the nuclei. Supernatant at this step was collected as cytoplasm extract. Nuclei were washed 1-2 times in the homogenization buffer, collected by centrifugation and resuspended in 430 mM sucrose, 70 mM KCl, 2 mM $MgCl_2$, 10 mM Tris-HCl (pH 8), 0.4 U/µl Murine RNase Inhibitor, 0.2 U/µl SUPERase-In, cOmplete Protease Inhibitor Cocktail (that optionally allowed storage of the prepared nuclei at -80°C). If needed, the nuclei were strained using 40 µm Flowmi strainers (Sigma). For estimation of nuclei concentration, Hoechst DNA dye or propidium iodide was added and nuclei were counted on Countess II FL Automated Cell Counter (Thermo Fisher Scientific). Hoechst-positive nuclei from adult human vermis, flocculonodular lobe and deep nuclei were sorted by flow cytometry to remove cellular debris present in preparations from white matter-rich brain tissues. The nuclei were sorted in PBS on BD FACSAria III using BD FACSDiva 8.0.1 software (BD Biosciences). Gates were set on FSC/SSC and at the excitation wavelength of 405 nm (Extended Data Fig. 1b). The obtained suspension contained 68-86% Hoechst-positive particles, as determined on Countess FL Automated Cell Counter. A few modifications in the nuclei preparation method were applied in the pilot phase of data production: (1) the tissue was lysed in HEPES-based homogenisation buffer (250 mM sucrose, 25 mM KCl, 5 mM $MgCl_2$, 10 mM HEPES (pH 8), 0.1% IGEPAL, 1 µM DTT, 0.4 U/µl Murine RNase Inhibitor, 0.2 U/µl SUPERase-In), the nuclei were fixed with DSP (dithio-bis[succinimidyl propionate]) as described[58] and then processed as

above; (2) the tissue was lysed in Triton X-100 homogenisation buffer (320 mM sucrose, 5 mM $CaCl_2$, 3 mM Mg-acetate, 10 mM Tris-HCl (pH 8), 0.1 % Triton X-100, 2 mM EDTA, 0.5 mM EGTA, 1 mM DTT, 0.4 U/µl Murine RNase Inhibitor, 0.2 U/µl SUPERase-In). The two mouse datasets produced with the modified protocols were comparable to the ones produced with the standard protocol in terms of cellular composition and gene expression (Extended Data Fig. 1d) and were therefore included in the merged datasets. Supplementary Table 1 lists nuclei preparation details for each sample.

RNA extraction and sample quality control.

Cytoplasm extracts or nuclei suspensions were mixed with 40 mM DTT-supplemented RLT buffer (Qiagen) and 100% ethanol at 2:7:5 ratio. The mixtures were subjected to RNA purification with RNeasy Micro Kit (Qiagen). RNA quality numbers (RQN), determined on Fragment Analyzer (Advanced Analytical), were above 7 for all samples except some of the human infant and toddler samples, and opossum P42 and adult samples (Supplementary Table 1).

Library preparation and sequencing.

Chromium Single Cell 3' Reagent kits (v2 or v3 chemistry) and the Chromium Controller instrument (10x Genomics) were used for single cell barcoding and library construction according to manufacturer's protocols. In most of the experiments 15,000 nuclei were loaded per channel (range 13,000-17,000). cDNA was amplified in 12 PCR cycles. Libraries were quantified on Qubit Fluorometer (Thermo Fisher Scientific) and the average fragment size was determined on Fragment Analyzer. Libraries were sequenced on Illumina NextSeq 550 (26/28 cycles for Read 1, 8 cycles for i7 index, 57/56 cycles for Read 2 in case of v2/v3 libraries) or HiSeq 4000 (26/28 cycles for Read 1, 8 cycles for i7 index, 56-74/74 cycles for Read 2 in case of v2/v3 libraries).

Genome annotations.

For human and mouse we used gene annotations from Ensembl release 91[59], based on reference genome assemblies hg38 (human) and mm10 (mouse). For opossum, we used monDom5-based annotations from Ensembl release 87 extended using stranded poly(A) RNA-seq datasets[19] from the adult brain as described[60]. We split each of the opossum chromosomes 1 and 2 at the nucleotide position 536,141,000, which does not overlap any gene, given that the large size of these chromosomes is not compatible with many bioinformatics pipelines. We generated custom genome reference files with *cellranger mkref* pipeline from Cell Ranger (10x Genomics). Since snRNA-seq captures both mature mRNA and unspliced pre-mRNA, we additionally created pre-mRNA references by merging exons and introns of each gene. Orthology relationships were extracted from Ensembl release 91. The list of mouse transcription factors was downloaded from the animal TFDB (v.3.0)[61].

General data processing and quality control.

Raw sequencing data were demultiplexed using *cellranger mkfastq* pipeline (Cell Ranger versions 2 or 3.0.2, 10x Genomics, internally calling *bcl2fastq*). Read alignment and counting of Unique Molecular Identifiers (UMIs) was performed with *cellranger count*. Reads from each library were aligned and counted in two modes: (1) using the mature mRNA reference (exons) or (2) the pre-mRNA reference (exons and introns). We used the pre-mRNA counts (exons and introns) in most of the analyses to maximise the amount of reads for quantification. We used the mature mRNA counts (exons) in specific cases: library-level quality control (Extended Data Fig. 1d) and presence/absence expression differences (Fig. 4e-i, Extended Data Fig. 11-12). Valid barcodes (i.e. droplets containing a nucleus) were identified by leveraging the higher abundance of intronic reads (originating from pre-mRNAs) in nucleus-containing droplets compared to empty droplets that mostly contain background levels of cytoplasmic mature mRNAs. For each barcode the fraction of intronic UMI counts was estimated as [1-(exonic counts/pre-mRNA counts)]. Barcodes were clustered by their fraction of intronic UMIs using a Gaussian mixed model (mclust[62] package v5.4.3, R) with two expected clusters. The

barcodes in the cluster with higher mean fraction of intronic reads were considered as valid. Duplicates were removed using Scrublet[63] (v0.2, python v3.6.8) by calculating the 'doublet score' for each barcode and removing barcodes with a 'doublet score' higher than the 90% quantile. One human sample (SN296) was removed during the cell type annotation procedure due to high contamination from neighbouring brain regions (see below). Altogether, 115,282 mouse, 180,956 human and 99,498 opossum cells, with a median of 2,392 UMIs per cell, passed the filtering steps (Extended Data Fig. 1a,e).

To assess the quality of the sequenced libraries, we aggregated expression values (mature mRNA counts) across all cells within each batch into pseudobulks and calculated Spearman's rho correlation coefficients between the pseudobulks using genes expressed in at least 10% of the cells in at least one biological replicate (human n = 7,696; mouse n = 4,806; opossum n = 2,765). We observed high correlations between the libraries from the same developmental stage, even when different Chromium reagents (v2 and v3) were used to produce the libraries (Extended Data Fig. 1d).

Data integration and clustering.

We used LIGER (0.4.2, R)[14] to perform batch correction and integrate data across stages within each species. Libraries across all developmental stages and individuals were considered as individual batches. Normalization and selection of highly variable genes were performed using LIGER with default parameters, followed by integrative non-negative matrix factorization (*optimizeALS* function) with k=100. The obtained embeddings were then used as the basis for UMAP visualisation (uwot 0.1.10, R)[64]. To annotate cell types, we applied iterative unsupervised clustering within each species using the SCANPY (v1.5.1) implementation of the Louvain algorithm with a resolution of 3. First, we clustered the entire datasets for each species. For each identified cluster we split the data by batch and repeated the LIGER integration (k=50) and Louvain clustering as described above to divide the data into subclusters. Batches with low cell contribution (<50 cells) to a cluster were excluded from sub-clustering. This iterative procedure yielded 68 (human), 61 (mouse) and

67 (opossum) clusters split into 574-611 subclusters for each species (Supplementary Tables 2-4, Extended Data Figure 2b).

We integrated the snRNA-seq data across species and across developmental stages in a common embedding (in pairs or all three species combined) using LIGER as described above. We used 1:1 orthologous genes detectable (i.e. at least 1 UMI) in all batches and variable across cells (n=6,101 for mouse/human; n=5,019 for mouse/opossum; n=3,742 for global). The initial integration resulted in manifolds where species-specific differences were still visible. To further merge the embeddings across species, MNN-correct[65] (*fastMNN*, R, *batchelor package 1.0.1*) was applied with species assignment as the integration vector. This generated 100-dimensional aligned embeddings that were used for transfer of cell type labels (pairwise embeddings) or estimation of pseudoages (global embedding) as described below. For visualisation purposes, we used the aligned embeddings to compute 2D and 3D UMAP coordinates for the cross-species integrated datasets (uwot 0.1.10, R[64], Extended Data Fig. 2b,d; https://apps.kaessmannlab.org/sc-cerebellum-transcriptome).

Cell type annotation and label transfer.

Given that the development of cerebellar cell types and their marker genes have been mostly described in mouse[6,12,7,66], we first annotated the cells in the merged mouse dataset. Besides literature-based marker genes, we extensively used the *in situ* hybridisation data from the Allen Developing Mouse Brain Atlas[15] and GenePaint[67,68]. We assigned each subcluster to a broad lineage (e.g. *VZ* for neurons born at ventricular zone), cell type (e.g., *Purkinje*) and cell state (e.g., *Purkinje_defined*). Cell states that displayed remaining variability were further split into subtypes (e.g., *Purkinje_defined_FOXP1*; Extended Data Fig. 2a-c). The cells that were not included in any subcluster were annotated only in case their identity could be unequivocally determined (Supplementary Table 2). The smallest clusters (<100 cells) were annotated as whole clusters. Although we mostly use the term "type" to group cells committed to a distinct mature cell fate and "state" to refer to differentiation status that often form a continuum within each cell type category (Extended Data Fig. 2a,c), there are a few notable exceptions: (1) splitting early neuroblasts based on their final fate was often not

possible and we therefore included separate "cell type" level categories (e.g., *VZ_neuroblast*) to label these nascent postmitotic neurons; (2) the "cell type" level category *GC/UBC* was used to annotate subclusters that displayed co-expression of granule and unipolar brush cell markers; (3) we split neural progenitors into spatiotemporal "subtypes", although some of these might alternatively be considered as sub-states of the same progenitor subtype (e.g., *progenitor_RL_early* and *progenitor_RL*); (4) in case of the *oligodendrocyte_progenitor* cell state the sub-states (*pre-OPC*, *OPC* and *COP*) were distinguished at the level of "subtype" categories; (5) we grouped all immune cells into a single "cell type" category, because of low numbers of these cells in our datasets. The terms used to designate cell state categories include descriptors "progenitor" (proliferating cells), "neuroblast" (nascent postmitotic neurons), "glioblast" (glial cells exiting cell cycle), "differentiating", "defined", and "maturing" ordered from less differentiated to more mature cell states, but not formally aligned across different cell type lineages (Extended Data Fig. 2a,c). In sum, 97% of the cells in the mouse dataset were specified at the level of cell state, out of which 51% were additionally assigned to a subtype (Supplementary Table 2). 1.0% of cells were annotated only at the level of cell type, these included low-quality granule cells and unresolved subclusters that contain cells from different cell types located at the nuclear transitory zone (*NTZ_mixed*).

Next, we used the pairwise cross-species aligned embeddings to transfer the annotation labels from the mouse subclusters to the subclusters in the human and opossum datasets (Extended Data Figure 2b). We determined centroids of all species-specific subclusters in the aligned embeddings, calculated centroid Pearson correlations and transferred the label of the highest correlating mouse subcluster to each human and opossum subcluster. We curated the transferred labels by inspecting the expression of marker genes in each subcluster in the human and opossum datasets (Supplementary Tables 3-4). In the human dataset we noticed two clusters (23 and 34) that mostly contained cells from one batch (SN296) and where expression of *HOX* genes was detectable, indicating that these clusters contained contaminating brainstem cells. We removed this batch from the human dataset and did not annotate the remaining cells (n=113) in the two clusters. Out of the transferred subcluster labels, 69/61% (human/opossum) were confirmed, 4.2/9.9% were re-annotated because of sampling

differences (e.g., midbrain-derived *MB_neuroblast* detected in the opossum dataset only), 23/19% of the labels were adapted at the level of subtypes or related cell states, 1.7/4.7% of the labels were changed at the level of cell type, and 2.8/4.8% of the labels were removed because of unclear identity of the cells in these subclusters (mixed populations of cells, likely doublets and/or low quality cells). The human and opossum subclusters that did not receive a transferred label, were annotated *de novo* as described for the mouse dataset. As a result of the label transfer and curation procedures 97% and 94% of the cells in the human (not considering the removed brainstem library) and opossum datasets were specified at the level of cell state, out of which 47% and 40%, respectively, were additionally assigned to a subtype (Supplementary Table 3-4). 1.1% of human cells and 2.6% of opossum cells belonged to subclusters that contain cells from different cell types located at the nuclear transitory zone (*NTZ_mixed*). 0.6% of human cells belonged to subclusters that contain glutamatergic neurons with uncertain identity originating from adult deep nuclei-enriched samples, and expressing markers of deep nuclei neurons (*NDUFA4*, *FAU*, *ATP5E*) and granule cells (*PAX6*, *SLC17A7*; *glutamatergic_uncertain*). The unlabelled cells (2.5% of mouse, 1.2% of human and 3.7% of opossum cells) were included in the merged datasets, but excluded from downstream analyses of cerebellar cell type transcriptomes.

Some annotation categories were not detected in all three species (Extended Data Fig. 2c). Out of these, many involve contaminating cell types located at the dissection borders: *progenitor_RP* (roof plate), *motorneuron*, and *neural_crest_progenitor* groups detected only in mouse, *progenitor_MB* and *MB_neuroblast* (midbrain) detected in opossum, *isthmic_neuroblast* detected in human and opossum, and *GABA_MB* (midbrain) detected in human. Some categories were not detected in human likely due to their overall low numbers: *isth_N_SLC5A7* is the least abundant subtype among the isthmic nuclei neurons in the mouse and opossum datasets (less than 250 cells) and these cells are situated at the dissection border; ependymal cells have low abundance in the mouse (57 cells) and opossum (160 cells) datasets, and are likely present in the human subcluster "orig.cl_38_9" (Supplementary Table 3) that, however, also contains cells from other types and was therefore not assigned to a cell type; *MBO* (midbrain-originating cells) were not distinguished in the

human dataset, but markers of this cell type were expressed among some of the cells annotated as *NTZ_mixed* ("orig.cl_16_1", Supplementary Table 3). Sampling of tissue fragments in human could be the reason why we did not distinguish *GC_diff_2_OTX2* in this species. The lower resolution of Purkinje subtype mapping in human could be related to the differences in Purkinje cell sampling, developmental dynamics, and/or subtype prevalence. In the mouse dataset, we did not distinguish subtype *progenitor_VZ_anterior*, likely due to limited resolution, given that it was possible to identify this subpopulation based on snATAC-seq data[25]. We also did not detect *preOPC*s in the mouse dataset: this population was mostly sampled from a single stage in human (17 wpc) and opossum (P14), and is in general expected to be scarce in the cerebellum given that more than 90% of oligodendrocytes in the mouse cerebellum originate in the ventral hindbrain[69]. The presence of group *glutamatergic_uncertain* in the human dataset only is due to inclusion of data from adult samples microdissected from deep nuclei region. Similarly, inclusion of these samples could underlie the unique detection of oligodendrocyte progenitors subtypes *OPC_early* and *OPC_late* only in the human dataset. Nevertheless, separation of human OPCs into subtypes is supported by previous studies[70]. It remains unclear if the human-unique categories *glut_DN_maturing*, *GC_diff_1_early* and *GC_diff_1_late*, and the opossum-unique categories *interneuron_MEIS2* and *ependymal_progenitor* are a result of biological or technical variation.

External sc/snRNA-seq datasets

Processed data and annotations of adult mouse cerebellum snRNA-seq dataset by Kozareva *et al.*[7] were downloaded from https://singlecell.broadinstitute.org/single_cell/study/SCP795. Processed data and annotations of human fetal cerebellum sn/scRNA-seq dataset by Aldinger *et al.*[13] were downloaded from https://www.covid19cellatlas.org/aldinger20/. Cell type annotations of mouse developing cerebellum scRNA-seq dataset by Smith *et al.*[21] were downloaded from https://www.ncbi.nlm.nih.gov/geo/query/acc.cgi?acc=GSE209915. Cell type annotations of mouse developing cerebellum scRNA-seq datasets by Carter *et al.*[10] and by Vladoiu *et al.*[11] were provided by the

authors through personal communication. Processed data of human embryonic and fetal (6.6, 6.7, 6.9, 8, 8.1, 8.5 11.5, 12, and 14 wpc) cerebellum scRNA-seq data by Braun *et al.*[22] were downloaded from https://github.com/linnarsson-lab/developing-human-brain. We used Seurat[71] v4.0.6 to transfer the cell type annotations from our human dataset to the Braun *et al.*[22] cerebellum data. For each dataset, gene expression counts were scaled to 10,000 and log-normalised. After identifying the 5000 most highly variable genes for each dataset, we used their intersection (2694 genes) to identify anchors between the datasets with the function FindTransferAnchors(). Annotations were then transferred from our dataset (reference) to Braun *et al.*[22] (query) with the function TransferData() with k.weight = 30 and weight.reduction = "cca".

From the Aldinger *et al.* dataset, we extracted barcodes annotated as Purkinje cells (n=25 711), and used LIGER[14] (k=10) to perform batch correction and integrate data across stages, assigning batches by *sample_id* and *experiment*. We performed Leiden clustering (leidenAlg[72], version 1.0.5; resolution 0.6), and identified clusters that contained barcodes that likely represent doublets or contaminated nuclei, based on the higher number of counts in these clusters and co-expression of markers of several cell types (*PAX2* for interneurons, *PAX6* for granule cells, *SOX2* for astroglia lineage cells). After excluding the contaminated clusters, we performed LIGER integration (k=10) and Leiden clustering (resolution 1.2) on the remaining barcodes (n=14,246, 55% of the barcodes annotated as Purkinje cells in the original dataset). We annotated 15 of the obtained 17 clusters based on the expression of Purkinje subtype markers *RORB*, *FOXP1*, *CDH9*, *ETV1*, *EBF1* and *EBF2*. 732 barcodes (2 clusters, 5.1% of the barcodes) were not assigned to a subtype.

Quantification of cell type abundances and ratios

To quantify the relative abundances of cell types at each developmental stage in mouse, human and opossum, we grouped cells by cell type and required at least 50 cells per group (this resulted in the removal of 0-280 cells per developmental stage). For Fig. 1e, we quantified the cell type proportions across the whole datasets. The human adult deep nuclei libraries were excluded from Fig. 1e, since we did not sample deep nuclei separately in the two other species. The cell type proportions in individual adult human samples from different

cerebellar cortical regions and deep nuclei are shown in Extended Data Fig. 7c. For Fig. 1f, we quantified the cell type proportions in each biological replicate separately (human 8 wpc libraries from the same individual produced with the same Chromium version were merged; human adult deep nuclei libraries were excluded), and determined the median between the biological replicates. In Fig. 1f, we did not consider cells that were not assigned to a cell type or belong to cell types/subtypes, which are from brain regions adjacent to the cerebellum: *GABA_MB*, *progenitor_MB*, *progenitor_isthmic*, *motor_neuron*, *neural_crest_progenitor*, *isthmic_neuroblast*, *MB_neuroblast*.

For Extended Data Fig. 7f, we estimated the proportions of Purkinje cells using the cell type annotations provided by the original studies, if available. For Carter *et al*. mouse dataset, we additionally included cells originally annotated as *GABA Progenitor* among the Purkinje cell counts, as they express markers of differentiating Purkinje cells (*Lhx5*, *Foxp2*)[10]. For Vladoiu *et al*. mouse dataset, we additionally included cells originally annotated as *Brainstem progenitors* among the Purkinje cell counts, as our previous analyses indicated that these represent differentiating Purkinje cells[25]. For Braun *et al*. human dataset, cells with a transferred label *Purkinje* were included among the Purkinje cell counts.

For Fig. 2e, we calculated the ratio of cell numbers of the early-born Purkinje subtypes (FOXP1, RORB) and the late-born subtypes (CDH9, ETV1) in each biological replicate. We only included samples that met the following criteria: (i) they come from fetal stages when Purkinje cell generation is complete (9-20 wpc in human), (ii) the relative abundances of *Purkinje_maturing* state cells (which were not separated into subtypes) among the *Purkinje_defined* and *Purkinje_maturing* cells is below 5% (our dataset; Extended Data Fig. 3i), (iii) at least 50 subtype-assigned cells are present. In the case of the Aldinger *et al*.[13] dataset we excluded samples that only contained the hemisphere or vermis (n=2), which altogether resulted in the inclusion of 9 samples representing 8 fetal stages (2 replicates for 14 wpc, and 1 replicate for each other stage). For subtype relative abundances presented in Extended Data Fig. 3i,r, 4j and 5h, we required at least 50 cells of the respective cell states to be present in a sample.

Bayes modelling of cell type abundance differences

To test for differences in cell type abundances (Extended Data Fig. 7d), we applied a Bayesian hierarchical model that accounts for species-specific, biological and technical variability. For each species, stage and cell type the relative abundances were modelled using a binomial model. The true proportion of a given cell type within each species and stage was treated as an unobserved variable, which we modelled as a normal distribution to simulate the expected distribution of biological relative abundances of the given cell type. The mean of this normal distribution is drawn from a species and stage specific Student's T distribution with uninformative priors. The standard deviation is drawn from a wide exponential distribution with $\lambda = 1$. These settings set a broad prior to the model. Specifically, we modelled the number of cells of a certain cell type i as follows:

$$
\begin{aligned}
y_i &\sim Binomial(N, \alpha_i) \\
\alpha_i &\sim Normal(\alpha_{0i}, \sigma_i), 0 \leq \alpha_{0i} < \text{[illegible]} \\
\alpha_{0i} &\sim StudenT(1, 1.5, 1) \\
\sigma_i &\sim \exp(1), \sigma_i > 0
\end{aligned}
$$

where N is the total cell count and y_i represents the relative abundances of an investigated cell type within a distinct biological replicate. The hyperparameter α_i estimates the species-specific proportion of a certain cell type. α_i is sampled from a species and stage specific normal distribution. Only biological replicates with more than 50 cells of the target cell type were subjected to the fitting. The model was fitted using RStan[73] (v2.19.3, R, *sampling* function; *iter=4,000*, *control=list(adapt_delta=0.99)*). We then computed the pairwise differences of α_0 between the species, using the simulated posterior distributions. We evaluated the 95% highest density interval (HDI[74], *HDInterval*, v0.2.2, R) of the results and tested for differences using the Region Of Plausible Equality (ROPE) method. If the HDI of at least two comparisons (pairwise between the three species) did not overlap 0, we assumed a difference in cell type proportion.

Of note, although independently developed, our approach is similar to the method recently established by Phipson et al. 2022[75] (propeller) that performed well in an independent comparison of statistical methods for cell type composition analysis[76]. Bayesian framework was also implemented by Büttner et al. 2021[77]

(scCODA) and shown to improve the performance compared to other methods, particularly when only a few biological replicates are available. We opted not to use scCODA[77], since it requires selection of a reference cell type.

Identification of overdispersed genes.

To stratify for informative genes, we identified highly variable genes based on an overdispersion cutoff. This approach was used for the estimation of correspondences between developmental stages across species (see *Stage correspondences across species*), comparing cell states and subtypes between datasets (see *Cross-species correlations and comparisons to the adult mouse data*), and identification of genes that are dynamic during neuronal differentiation (see *Gene expression trajectories*). Gene expression was normalized by dividing the number of UMIs by the sum of UMIs per cell (size factor). Next, we calculated the mean expression and variance using the normalized values to compute the gene-wise variance to mean ratio (*VMR*). Assuming Poisson distribution of non-informative genes, we estimated the expected variance to mean relationship (pVMR) by averaging over the inverse size factor. We expected genes that are highly variable to exhibit:

$$VMR > \alpha\, pVMR$$

We set α, if not stated otherwise, to 1.5.

Pseudoages.

The 100-dimensional cross-species integrated global embedding of human, mouse and opossum datasets was used to determine a quasi-aligned continuous developmental vector, called pseudoage[18]. First, each mouse cell was assigned with a simple index of development, depending on its developmental stage, i.e. the first sampled stage (E10.5) was assigned with 1, the second (E11.5) with two. Next, this dataset was subsampled for each mouse time point to either 1,000 cells or the maximum number of cells per cell type, whichever value was smaller, to reduce the effect of cell type abundance differences between the stages. The resulting matrix was used as the reference. Next, for each cell in the human, mouse and opossum datasets, the 25 nearest reference

cells (mouse) were identified and the mean of their developmental index was assigned to each query cell as its pseudoage.

Stage correspondences across species.

To establish correspondences between the developmental stages sampled in mouse, human and opossum, we applied three different metrics: (I) transcriptome similarity, (II) pseudoage similarity, and (III) cell state abundance agreement.

(I) Transcriptome similarity. We identified the pairwise shared informative 1:1 orthologous genes in the human, mouse and opossum datasets (intersect of overdispersed genes, human vs. mouse n = 336, opossum vs. mouse n = 369 genes). We generated pseudobulks for each developmental stage by summing the UMI counts per gene from all cells. For each species, we normalized the expression values to CPM and mean-centered each gene's expression values. Next, we subsetted the normalized data for the pairwise shared informative genes, to calculate the Spearman correlation coefficients between the developmental stage pseudobulks from all species, and computed the correlation distances by taking the arccosine of the distance.

(II) Pseudoage similarity. We globally binned the cells from all species based on their pseudoages into 50 equally-sized bins. We then inferred for each developmental stage the proportions of cells in different pseudoage bins and used these proportions to calculate Manhattan distances for all cross-species developmental stage pairs.

(III) Similarity of cell state proportions. For each cross-species pair of developmental stages, we computed the proportion of cells that had the same cell state annotation, and used these proportions to determine the pairwise Manhattan distances.

We used the dynamic time warping algorithm (dtw[78], dtw v1.20, R) to find the lowest distance path through the 2D developmental plane in the distance matrices from all three approaches. We used mouse as a focal species for assigning correspondences. The three metrics showed good overall agreement (Extended Data Fig. 2e-g). When a developmental stage in human or opossum aligned with two or more stages in mouse, we kept

the one with the smallest transcriptome correlation distance (Extended Data Fig. 2e-g). We grouped human samples from Carnegie stages 18 and 19 (7 wpc) as both show low transcriptome correlation distance to E11.5 in mouse. The toddler stage in human and P60 stage in opossum best matched P14 and/or P63 stages in mouse, but were kept as a separate intermediate stage between P14 and P63 in mouse, given that mouse P14 was assigned to the infant stage in human and P42 in opossum, and mouse P63 was assigned to the adult stage in both human and opossum. We note that the estimated stage correspondences are dependent on the sampling scheme and, although they overall agree with previous studies[19], should not be interpreted as absolute best matches.

Histology.

For cryosections, whole heads (E14.5, P1, P4, P5, P21) or dissected cerebella (P42) of opossums were mounted in Tissue-Tek OCT compound (Sakura Finetek) and frozen in isopentane cooled to its freezing point. 12 µm sagittal cryosections were collected on SuperFrost Plus slides (Thermo Scientific), fixed in 4% PFA in PBS for 12 minutes, washed 3 times with PBS and incubated in 70% ethanol at 4°C overnight. The sections were rehydrated and permeabilized in 0.2% Triton X-100 in PBS for 20 minutes and stained with DAPI and NeuroTrace 530/615 red fluorescent Nissl (both 1:300 in PBS) from the BrainStain imaging kit (Thermo Fisher Scientific) for 20 minutes at room temperature. After washing with 0.2% Triton X-100 in PBS for 3x10 minutes, the sections were mounted in Prolong Diamond Antifade Mountant (Thermo Fisher Scientific). Stitched Z-stack fluorescence images were acquired on Olympus CellSens widefield microscope equipped with 10x 0.4 NA and 20x 0.75 NA objectives, and motorized XY-stage and Z-drive. Extended Focus Imaging projection was calculated using the cellSens software.

For FFPE sections, whole heads of P4 and P14 opossums were fixed in 4% formalin and transferred to 70% ethanol for storage. After decalcification with EDTA (25% v/v), the specimens were embedded in paraffin and sectioned at 6 µm using a sliding microtome (Leica SM2010 R). Sections were stained with Heidenhain's AZAN. Microphotography was done with a LEICA camera (DFC490) mounted on a ZEISS Axioskop

equipped with 1.25X and 2.5X objectives, employing the standard LEICA Application Suite (LAS X) for image capturing.

The mouse histology images are from the Allen Developing Mouse Brain Atlas[15]. Human images are from the HDBR Atlas[79–81].

Multiplexed single molecule *in situ* hybridisation data generation and analyses.

The spatial mapping was performed using an available 12-week post-conception human cerebellum sample provided by the Human Developmental Biology Resource (HDBR; UK). The cerebellum was dissected, mounted in Tissue-Tek OCT compound (Sakura Finetek) and frozen in isopentane cooled to its freezing point. 10 µm coronal cryosections were collected on Molecular Cartography coverlips (Resolve Biosciences). The probeset targeted 100 genes (Supplementary Table 6) manually selected based on our snRNA-seq data to cover markers of the cerebellar cell (sub)types and states, and selected genes with presence/absence expression differences between human and mouse (see *Presence/absence expression differences*). Among the latter we selected genes that are expressed in the human respective cell type in the 11-17 wpc developmental time window based on snRNA-seq data, and for which absence in the mouse respective cell type is supported by public *in situ* hybridisation data[15,16] (Supplementary Table 6). Molecular Cartography probe sets were designed and the data was produced as a service at Resolve Biosciences, Monnheim. smFISH data was visualised in Fiji using the Polylux plugin (V1.9.0., Resolve Biosciences).

We performed cell segmentation based on the detected transcripts considering joint likelihood of transcriptional composition and cell morphology using the Baysor[82] (v0.5.2) Julia (v1.6.4) library. The following parameters were set for each sample: "min-molecules-per-gene=1", "min-molecules-per-cell=3", "scale=25.0", "scale-std='25%'", "estimate-scale-from-centers=true", "min-molecules-per-segment=2". The sampling step of Baysor was run with "new-component-weight=0.2" and "new-component-fraction=0.3". Importantly, only segments that passed the 0.95 confidence level, as assigned by Baysor, were considered as cells. A custom R script was used to convert the Baysor output into a classical scRNA-seq raw dataset. The

resulting data was integrated with the 11 wpc human snRNA-seq data (excluding cells from categories *GABA_MB* and *NTZ_mixed*) using the Python (v3.9) library of Tangram[83] (v1.0.3). For imputation of metadata, Tangram function "project_cell_annotation" was applied with default parameters. We imputed metadata separately at two levels: cell type and precisest label, which combines cell states and subtype information. Each segmented cell was assigned to the highest-scoring metadata category.

For the presentation of imputed cell type labels in Fig. 1d and Extended Data Fig. 12f, we required at least 15 transcripts and an area of >500 pixels (9.522 μm^2) per segment, and filtered out outliers that had the transcript numbers or area larger than the sum of the 3rd quantile and 1.5 times the interquartile range (n_transcripts > ~140; area > ~7040 pixels (134 μm^2)). As a result we kept 87,140 (73%) of the initial 119,059 Baysor-estimated segments. The median metadata prediction score (Tangram) of the selected segments was 0.072 in case of cell type imputation and 0.050 in case of precisest label imputation. The prediction scores varied per group, for instance among the more abundant cell types (>1000 segments) the median score was the highest for the predicted Purkinje cell segments (0.084) and the lowest for GC/UBC segments (0.042). Similarly, among the precisest labels, the prediction score was the highest for the most abundant categories Purkinje_defined_EB (0.062) and Purkinje_defined_LB (0.046). Therefore, we standardised the prediction scores per group, and further filtered for the segments with a prediction score above the 1st quantile. As a result, we confidently assigned cell type labels to 65,355 (75%) of the 87,140 segments. For the predicted precisest labels presented in Fig. 2g (Purkinje subtypes), we required the segments to have a concordant cell type assignment (Purkinje cell). 36,531 (89%) of the 41,036 Purkinje cell segments had concordant and high confidence precisest label assignments, with 29,560 segments assigned as Purkinje_defined_EB and 6,971 as Purkinje_defined_LB.

Immunohistochemistry.

Mouse P7 brains were dissected, cut sagittally at the midline, fixed for 24 hours in 4% PFA at 4 °C, cryopreserved in 30% sucrose overnight at 4 °C, and embedded in Tissue-Tek OCT compound (Sakura

Finetek). Cryoblocks were stored at –80 °C. 10 µm sagittal sections were cut, collected on SuperFrost Plus slides (Fisher Scientific) and stored at -80 °C until use.

For indirect immunofluorescence, sections were blocked in 10% normal donkey serum (EMB Millipore) in PBS with 0.5% Triton X-100 for at least 30 minutes at room temperature. Primary antibodies were incubated overnight at room temperature in blocking solution. Sections were washed for 30 minutes with PBS with 0.5% Triton X-100, and incubated with secondary antibodies in blocking solution for 1 hour at room temperature. Tissue sections were mounted in ProLong Gold Antifade Mountant (Invitrogen). The primary antibodies were diluted as follows: mouse anti-HCRTR2 1:100 (R&D, Cat.No.: MAB52461), chicken anti-TBR2/EOMES 1:500 (Millipore, Cat.No.: AB15894), and rabbit anti-LMX1A (1:1000; Sigma Aldrich, Cat.No.: AB10533). The secondary antibodies used were Donkey anti-Mouse IgG (H+L) Alexa Fluor 488 (Invitrogen, Cat.No.: A21202), Donkey anti-Mouse IgG (H+L) Alexa Fluor 568 (Invitrogen, Cat.No.: A10037), Donkey anti-Rabbit IgG (H+L) Alexa Fluor 488 (Invitrogen, Cat.No.: A21206), Donkey anti-Rabbit IgG (H+L) Alexa Fluor 568 (Invitrogen, Cat.No.: A10042), and Goat Anti-Chicken IgY (H+L) Alexa Fluor 568 (Abcam, Cat.No.:ab175477).

For Immuno-SABER[84], primary antibodies were first conjugated using photo-crosslinking oYo-Linker oligonucleotides (AlphaThera Inc.) 1 µl of the oYo-Linker was mixed with 1 µg of primary antibody and incubated for 2 hours under UV light on ice. After conjugation, the oligo-tagged antibodies were stored under the same conditions as the unconjugated antibodies. Primer-exchange reaction (PER) was used to extend primers into ssDNA concatemers as described[85]. Briefly, a primer overlapping with the oligonucleotide crosslinked to the antibody, and the respective hairpin oligonucleotide was designed. Reactions were carried out in 100 µl of 1xPBS, 50 nM - 1 µM hairpin oligonucleotides in 10 µl IDT-TE buffer, 10 nM Clean.G hairpin (CCCCGAAAGT GGCCTCGGGC CTTTTGGCCC GAGGCCACTT TCG), 300 µM dATP, dCTP and dTTP (NEB), 10 mM $MgSO_4$, 400 U/ml Bst LF polymerase (McLab), and 1 µM primer. Before adding 10 µl of 10 µM primer solution to the reaction, the reactions (90 µl) were pre-incubated for 15 minutes at 37°C. The reactions were then incubated for 2 hours at 37 °C, followed by heat-inactivation of the polymerase

for 20 min at 80 °C. Concatemers were purified and concentrated using MinElute Kit (Qiagen) and suspended in 25 µl water. The length of the concatemers was evaluated on 1 % E-Gel™ EX agarose gels (Thermo Fisher, Cat.No.: G401001). Gels were imaged using the SYBR Gold channel on a Typhoon FLA 9000 scanner. The following primary antibodies were used: rabbit anti-HCRTR2 (Alomone Labs; Cat.No.: AOR-002), rabbit anti-TBR2/EOMES (Millipore; Cat.No.: ABN1687) and rabbit anti-LMX1A (Sigma-Aldrich; Cat.No.: AB10533). The oligonucleotides conjugated to the antibodies were: 5'- TTCAGTTCTT GTATCGCGTC ACTTATCGGT TATTGTCCTC TCGC-3' for anti-HCRTR2, 5'-TTGTTTCCTA TATTTAGCGT CCGTGTCGTT CTCCCGCGCA ACAG-3' for anti-LMX1A, and 5'-TTAACAATTC AGCTCCGCCT TATACCGTCT TACCGCCAAC ATCG-3' for anti-TBR2/EOMES. The extension primers were as follows: 5'-GCGAGAGGAC AATAACCGAT AAGTGACGCG ATACAAGAAC TGTTTTTATT CATTTACATT CATTTAC-3' for anti-HCRTR2, 5'- CTGTTGCGCG GGAGAACGAC ACGGACGCTA AATATAGGAA ACTTTTTACA TCATCATACA TCATCAT-3' for anti-LMX1A, and 5'- CGATGTTGGC GGTAAGACGG TATAAGGCGG AGCTGAATTG TTTTTTACT CTACTACACT CTACTAC-3' for anti-TBR2/EOMES. The hairpin oligonucleotides were as follows: 5'-ATTCATTTAC GGGCCTTTTG GCCCGTAAAT GAATGTAAAT GAA-3'/InvdT/ for anti-HCRTR2, 5'- ACATCATCAT GGGCCTTTTG GCCCATGATG ATGTATGATG ATG-3'/InvdT/ for anti-LMX1A, and 5'- ACTCTACTAC GGGCCTTTTG GCCCGTAGTA GAGTGTAGTA GAG-3'/InvdT/ for anti-TBR2/EOMES.

For Immuno-SABER stainings, cryoslides were thawed for ~5 minutes at room temperature, and washed twice in PBS. For permeabilization and blocking, the slides were incubated in blocking buffer containing 4 mM EDTA, 0.1% dextran sulphate, 0.5 mg/ml sheared salmon sperm, 100 µM random blocking oligos, 0.5% Triton X-100 and 10% normal donkey serum in PBS for 3x10 minutes. Primary antibodies were hybridised with three 14 nt oligos complementary to the crosslinked oligo-tag to reduce nonspecific binding. 0.2 µl of each 100 µM oligo was mixed with the conjugated primary antibody in 10 µl of 4 mM EDTA, 0.1% dextran sulphate, 0.5 mg/ml sheared salmon sperm, 0.5% Triton X-100 and 10% normal donkey serum in PBS, and rotated for 30 minutes at room temperature. Next, the antibody mixture was applied to the slides and incubated

overnight at 4 °C. The final dilution of the antibodies (respective to the original stock) was as follows: rabbit anti-HCRTR2 1:100, rabbit anti-TBR2/EOMES 1:1000, and rabbit anti-LMX1A 1:500. The slides were then washed once with blocking buffer (5 min), three times with 0.5% Triton X-100 and 10% normal donkey serum in PBS (a' 10 min), and two times with PBS (a' 5 min). Primary antibodies were then post-fixed using 5 mM BS3 (Thermo Scientific, Cat.No.: 21580) in PBS for 30 minutes at room temperature. After three washes with PBS (a' 5 min), 60% formamide was used to remove the 14 nt oligos on the primary antibodies. The formamide was exchanged every 3 minutes for a total of 12-15 minutes. After three washes with PBS (a' 5 min), PER concatemers were hybridised to the primary antibodies. PER concatemers were diluted 1:25 in PER buffer containing 20% formamide, 10% dextran sulphate, 0.1% Tween-20, 2x saline sodium citrate, 0.5 mg/ml sheared salmon sperm, and incubated for 1 h at 37 °C. Removal of leftover PER concatemers was done by washing with 0.5% Triton X-100 in PBS for 10 minutes at 37 °C, followed by washing in 45% formamide in PBS for 5 minutes wash at room temperature and three 10 minutes washes with 0.5% Triton X-100 in PBS at 37 °C. Fluorophore-labelled imagers (IDT) were incubated at 1 µM in 0.5% Triton X-100 in PBS for 30 minutes at room temperature in the dark. The following imagers were used: 5'-/ATTO565N/tt GTAAATGAAT GTAAATGAAT-3'/InvdT/ for anti-HCRTR2, 5'-/Alex647N/tt ATGATGATGT ATGATGATGT-3'/InvdT/ for anti-LMX1A, and 5'-/ATTO565N/tt GTAGTAGAGT GTAGTAGAGT-3'/InvdT/ for anti-TBR2/EOMES. DAPI was used to counterstain DNA, samples were mounted in SlowFade antifade Gold Mountant (Invitrogen), and the coverslips were secured with FixoGum (Marabu). Images were acquired using a Leica Thunder System widefield microscope with an HC PL APO 20x/0.8 DRY objective. The images were acquired as z-Stacks with 0.5 µm step. ATTO565N was visualised using a 546 nm laser and Alexa 647N was visualised using a 633-nm laser.

Gene expression scores.

To quantify the expression of a group of genes of interest (GOI), we calculated gene expression scores akin to the approach used by La Manno *et al.*[18]. UMI data was normalised by calculating counts per million (CPM),

and subsetted for the GOI. Next, we scaled the genes' expression vectors to mean zero and unit variance, we averaged the scaled expression of all GOI to compute the score and calculated its 0.01 and 0.99 percentile. These percentiles were used for capping the score to eliminate outliers. Values outside this boundary were assigned to the nearest accepted value. This approach was used to quantify the expression of genes related to cell cycle[18] (Extended Data Figs. 6g and 7a) or genes that gained expression in different cell types in the human lineage (Fig. 4g, Extended Data Fig. 12a,b).

Cell type lineage assignments for ventricular zone neuroblasts.

Although the VZ neuroblasts could not be separated into the associated terminal cell types (parabrachial and noradrenergic cells, GABAergic deep nuclei neurons, Purkinje cells, interneurons) based on clustering, we observed differential expression of known lineage-specific marker genes between developmental stages, in line with the known temporal order of the emergence of these cell types in the cerebellar ventricular zone[6,20]. We thus further split the mouse VZ neuroblasts based on their developmental stage: E10.5 cells were assigned to the parabrachial/noradrenergic cell type lineage, E11.5 cells to GABAergic deep nuclei neuron lineage, E12.5 and E13.5 to Purkinje cell lineage, and all other cells into interneuron lineage. In human, VZ_neuroblast_3 cells from Carnegie stage 18 were assigned to the parabrachial/noradrenergic cell type lineage, VZ_neuroblast_1 cells from Carnegie stages 18-19, VZ_neuroblast_2 cells from Carnegie stages 18-22 and VZ_neuroblast_3 cells from Carnegie stages 19-22 were assigned to the Purkinje cell lineage, and all other VZ neuroblasts to the interneuron lineage. In opossum, some VZ neuroblast subclusters could be assigned based on marker genes: cells in *LMX1A* and/or *LMX1B* positive subclusters (orig.cl_27_3, orig.cl_39_0, orig.cl_39_1, orig.cl_39_6) were assigned to the parabrachial/noradrenergic cell type lineage, and cells in *PAX2* and/or *SLC6A5* positive subclusters (orig.cl_23_9, orig.cl_26_10, orig.cl_31_2, orig.cl_31_4, orig.cl_31_5) to the interneuron lineage. The rest of opossum VZ neuroblasts were split based on their developmental stage: E14.5 and P1 cells were assigned to the GABAergic deep nuclei neuron lineage, P4 and P5 cells to Purkinje cell lineage, and P14 and P21 cells to the interneuron lineage. These assignments

were used to visualize the VZ cell type lineages and to study the expression patterns of cell type marker genes amongst the VZ neuroblasts (Extended Data Fig. 3g,h), to perform LIGER integration for Purkinje and interneuron cell type lineages within each species (Fig. 2a, Extended Data Fig. 3o), and to integrate cells in Purkinje cell type lineage across species (Fig. 3d, Extended Data Fig. 9a; see also *Pseudotemporal ordering*).

Integration of data by subsets.

For visualization purposes we integrated data by different subsets of cells belonging to a broad lineage, to a cell type lineage, or to a developmental stage. In this analysis, interneurons from adult human deep nuclei samples (n=57) were not included in the human interneuron subset, given that these were low in numbers, co-clustered with the molecular layer interneurons, and were not distinguished in adult mouse and opossum datasets, for which we only sampled whole cerebella. The integration of different subsets was performed as described for the whole datasets (see *Data integration and clustering*) using LIGER[14]. The number of components used for integrative non-negative matrix factorization (*optimizeALS* function) was as follows: Purkinje cell type lineage in mouse k=70, human k=50, opossum k=70 (Fig. 2a); interneuron cell type lineage in mouse k=15, human k=20, opossum k=20 (Extended Data Fig. 3o); VZ broad lineage in mouse k=40, human k=50, opossum k=30 (Extended Data Fig. 3c,h); RL/NTZ broad lineage in mouse k=15, human k=15, opossum k=15 (Extended Data Fig. 4c,g); RL/EGL broad lineage in mouse k=30, human k=40, opossum k=40 (Extended Data Fig. 5c,f); glia broad lineage in mouse k=30, human k=40, opossum k=70 (Extended Data Fig. 6d). For the integration of individual developmental stages (Extended Data Figs. 3j, 7b) we used k=25.

Cross-species correlations and comparisons to the adult mouse data.

To match cell states and subtypes across species, we calculated Spearman correlation coefficients for all cross-species pairs within a subset (a broad lineage or a cell type). Interneurons from adult human deep nuclei (n=57) were excluded from this analysis (see also *Integration of data by subsets*). We required at least 50 cells, and subsampled up to 1000 cells per category. We used only the 1:1 orthologous genes that were overdispersed within a subset in each of the three species (i.e. intersect of highly variable genes). The number of the used

genes for each subset is specified in the figure legends (Fig. 2d, Extended Data Figs. 3d,t, 4d,k, 5d,g, and 6e,f). Importantly, these analyses should not be used to make conclusions about the differences in evolutionary conservation of the individual categories, given that the comparisons are limited to the intersect of highly variable genes, and might be affected by imperfect matching between the developmental stages we sampled as well as between the individual subtype/state categories defined in each species. The latter is especially true for the cell states that form a differentiation continuum as we cannot exclude slight shifts in the borders of these categories between the species.

We used the same approach to match mouse Purkinje and granule cell developmental subtypes defined in this study to the adult subtypes reported by Kozareva *et al.*[7], using all mouse genes that were overdispersed in both datasets (i.e. intersect of highly variable genes; Extended Data Figs. 3m,s, and 5k). We note that the adult mouse dataset[7] was produced by methods similar to the ones used in this study: nuclei were extracted from frozen tissue samples dissected from different lobules of the cerebellar cortex and the libraries were prepared using v3 Chromium reagents (10x Genomics).

Principal components analysis.

To evaluate global relationships among the cell type-specific transcriptomes during development in the three species, we performed principal components analysis (PCA) using three-way 1:1 orthologous genes. Cells originating from the same biological replicate and assigned to the same cell type were merged into pseudobulks by summing up the UMI counts per gene. Only pseudobulks that contained at least 150 cells were considered. Only genes which were expressed in at least 10% of cells in a single pseudobulk in any species and showed variability in all species (variance(CPM) > 0) were kept for downstream analysis. The data was normalized to CPM and gene expression was median-centered within each species and aggregated. The first 15 principal components of the merged dataset were approximated using *prcomp_irlba* (irlba[86] v2.3.3, R).

Marker gene identification.

To identify genes enriched in different cell states, term frequency - inverse document frequency (TF-IDF) transformation was applied (quickMarker function from the R package SoupX)[87]. *P*-values were computed using a hypergeometric test and corrected with the Benjamini Hochberg method. The count matrix was binarized with a threshold of > 0 UMI per cell. For each gene its expression within a specific group of cells was contrasted against all other cells in the dataset.

Conserved marker genes were called using an adjusted dataset. To reduce the effect of sampling differences between species, the data was aligned between the species: for each cell state, only those matched developmental stages were used, where at least 50 cells were present in all three species. Next, each cell state per matched developmental stage was sampled to 1,000 cells, if needed the group was randomly upsampled. This dataset was then used for marker gene identification as described above. Conserved markers were defined as genes that are enriched in all species in the same cell state with a corrected *P*-value of less than 0.01, display cell state-specific enrichment of > 2 and show expression (UMI > 1) in at least 10% of the cells. Conserved markers were ranked by maximum scaling the TF-IDF value per species and calculating a score as follows:

$$S_j = \sqrt{t_{j,Human}{}^2 + t_{j,Mouse}{}^2 + t_{j,Opossum}{}^2},$$

where t is the scaled TF-IDF value and S the ranking score per gene j.

Pseudotemporal ordering.

We extracted from all three datasets the cells that were assigned to the Purkinje cell (Extended Data Fig. 3g,h) or granule cell (Extended Data Fig. 5c) lineage. We only used the three-way 1:1 orthologous genes that were detectable (number of UMIs > 0) in all species. We aligned the data from all species in the low dimensional space of 50 principal components using the Harmony pipeline[88] (v1.0). Library and species identities were used as covariates. Next, we chose a starting cell, based on the UMAP embedding, and applied the SCANPY implementation of diffusion pseudotime[37] (DPT) algorithm. Specifically, we used the Harmony-corrected principal components as input for the diffusion map algorithm and projected the cells into a ten-dimensional

diffusion map, which together with the previously selected root cell was used as an input for the estimation of DPT values. Of note, the UMAP embedding of granule cells (Fig. 3d, Extended Data Fig. 9a,c) shows a gap between the differentiating and defined granule cells but this 2D representation does not influence the estimation of DPT values in the ten-dimensional diffusion map.

Gene expression trajectories.

We used the cross-species aligned pseudotemporal cell orderings to model gene expression trajectories in all three species. First, we split the pseudotime vector into ten equally sized bins. For each bin, pseudobulks consisting of cells from the same biological replicate were merged by summing up the UMIs per gene, only considering the replicates where the number of cells was at least 50. Next, we determined for each pseudotime bin and species the mean UMI counts between the biological replicates. To identify the genes that are dynamic during neuronal differentiation, we filtered for highly variable genes (as described in *Identification of overdispersed genes*) using the pseudotime binned UMIs. In this case we set α in the highly variable gene formula (see above) to 1. Selection was done per species and the intersection of dynamic genes across species was used in the next steps. The count matrix was normalised, using all genes, to counts per million (CPM), subsetted for the highly variable genes and combined such that each orthologue was added as an individual feature, i.e. each gene appeared three times in the matrix, one time for each species. To infer groups of genes with similar trajectories, we used the soft clustering algorithm Mfuzz[89] (v2.44.0) and clustered the genes into eight trajectory clusters. For each orthologue we determined its center of mass to infer its most prominent expression time window (Extended Data Figs. 9g, 10a). To order the trajectory clusters, we calculated the mean center of mass of all confident cluster members (cluster membership score > 0.5). The similarity of orthologues' trajectories was tested by calculating a similarity *P*-value from the cluster membership score:

$$p(x,y) = \sum_{i=1}^{k} m_{xi} m_{yi},$$

where m_{xi} and m_{yi} is the cluster membership score for cluster i for the given orthologues from two species (x and y). The resulting P-value was adjusted for multiple testing using Benjamini-Hochberg method. Based on a set of rules, the orthologues were classified in multiple classes:

$$class \begin{cases} \text{x specific,} & \text{if } p(x,y) < 0.05 \wedge p(x,z) < 0.05 \wedge p(y,z) > 0.5 \wedge c_y = c_x \\ \text{preserved,} & \text{if } c_x = c_y \wedge c_x = c_z \wedge c_y = c_z \wedge p(x,y) > 0.5 \wedge p(y,z) > 0.5 \\ \text{diverse,} & \text{if } p(x,y) < 0.05 \wedge p(x,z) < 0.05 \wedge p(y,z) < 0.05 \\ \text{intermediate,} & \text{otherwise} \end{cases}$$

where x, y, z represent the studied species in any given combination, $p(x, y)$ is the adjusted P-value for the comparison of the given orthologues in species x and y, c_x the cluster with the maximum membership score for the studied orthologue in species x (human-specific, mouse-specific, marsupial). If one or more of the three orthologues did not reach a maximum membership score above 0.5, the gene was excluded from downstream analysis. Orthologous genes with either species-specific or diverse trajectories were grouped into the 'diverged' class (Fig. 3g).

We additionally measured the similarities between the trajectories of the orthologous genes by computing dynamic time warping distances (dtw[78] v1.20, R). Comparison of these distances for the groups of genes with either preserved, intermediate or diverged trajectories corroborated the clustering-based classification (Extended Data Fig. 9h). We further assessed the maximum and minimum pairwise dynamic time warping distances between the trajectories of the orthologues, to provide a quantitative measure of the amount of change for each gene (Figure 4c, Extended Data Fig. 10c).

The patterns of trajectory changes during Purkinje or granule cell differentiation were visualised using alluvial plots (ggalluvial[90] v0.12.3, R). For both cell types, each orthologous gene was mapped to its trajectory cluster in each species, and coloured according to its trajectory cluster in human (Fig. 4a, Extended Data Fig. 10b).

Presence/absence expression differences.

To assess presence and absence of gene expression in cerebellar cell types, we generated pseudobulks by summing up exonic UMI counts (mature mRNA reference, expressed orthologous genes) per biological replicate, and cell type (astroglia, oligodendrocytes, GABAergic deep nuclei neurons, Purkinje cells,

interneurons, glutamatergic deep nuclei neurons, granule cells, UBCs, NTZ neuroblasts, isthmic nuclei neurons, NTZ mixed cells, VZ neuroblasts, parabrachial cells, meningeal cells, and immune cells). For the meningeal and immune cell types, we summed up the counts from all cells in the dataset, because of their low abundance. We used the mature mRNA counts in this analysis, since most of the intronic reads arise due to internal priming from intronic polyA and polyT sequences[91] that can differ between the species. For each cell type, we only considered those matched developmental stages, for which there was data from all three species; and only those pseudobulks that had at least 50 cells. We normalized the data to CPM. In each species, we required a gene to reach a cutoff 50 CPM in at least 2 pseudobulks of a given cell type (or the single pseudobulk generated for meningeal or immune cells), to consider it as reliably expressed (Extended Data Fig. 11a). For genes failing to meet this cutoff, i.e. not reliably expressed in any of the cell types, we also considered their expression in bulk RNA-seq RPKM-normalized data covering overlapping stages of cerebellum development in the same species[19]. Genes not detected as reliably expressed in the cerebellum (in any cell type) based on our snRNA-seq data but reaching a RPKM value above 5 in the bulk RNA-seq data, were assumed to be affected by technical artefacts of the snRNA-seq measurements. In these cases, we removed the orthologous gene group from the downstream analysis. We further considered possible biases stemming from evolutionary divergence of the sequences and differences in gene annotation quality between the species (GC content, length), which could affect the efficiency of reverse transcription, PCR-amplification, and/or mapping of the sequences. We determined the maximum expression of each gene across all pseudobulks in each species, and compared the cross-species fold differences in gene's maximum expression to differences in gene length and GC content. We did not observe any strong correlation between fold difference in a gene's maximum expression and fold difference in its median-adjusted exonic length (human-mouse Pearson's r=0.07, human-opossum r=0.11, mouse-opossum r=0.06), 3'UTR length (human-mouse r=0.07), exonic GC content (human-mouse r=-0.01, human-opossum r=-0.01, mouse-opossum r=-0.02) or 3'UTR GC content (human-mouse r=0.01). Nevertheless, in our initial analyses we observed that genes we called as expressed in a species-specific manner tend to be longer (exonic length) in the species where expression is present compared to their

orthologous in species where expression is absent, indicating some length bias in detection sensitivities in the Chromium snRNA-seq data, as also noted previously[92]. Therefore, to reduce the effects of these biases in our cross-species comparisons, we removed the orthologous gene groups that show major differences in exonic length between the species. For this, we first calculated pairwise median-adjusted differences in exonic lengths of orthologous genes. Median-adjustment is necessary to consider global differences in exonic length between the species (global shifts are accounted for by the CPM normalisation). We only considered orthologous gene groups for which the pairwise adjusted exonic length fold differences were less than 3 (Extended Data Fig. 11a). In the comparative analyses of all three species (human, mouse, opossum), 5,693 genes were affected by the technical limitations related to gene detection and/or annotation in at least one of the species and removed, whereas 7,062 1:1 orthologous genes were kept in the downstream analysis (Extended Data Fig. 11g). In a separate comparison of human and mouse only, 4,135 genes were removed because of these technical limitations, and 8,620 genes were kept in the downstream analysis.

We then focused on the main glial and neuronal cell types in the cerebellum (astroglia, oligodendrocytes, GABAergic deep nuclei neurons, Purkinje cells, interneurons, glutamatergic deep nuclei neurons, granule cells, UBCs) to evaluate presence/absence of expression of three-way 1:1 orthologous genes. If a gene's expression in two pseudobulks within a matched developmental stage reached the cutoff of 50 CPM, we assumed the gene to be reliably expressed in this cell type (Extended Data Fig. 11a). Next, in each species we determined the maximum expression of each gene per cell type across all matched stages, and calculated the ratio to overall maximum expression within the species, and pairwise fold differences between the orthologues from different species (Extended Data Fig. 11a,b). The within-species comparison serves to reduce the effects of possible biases in the detection sensitivities between the species. We called a gene “present”, when it was reliably expressed in the cell type and its maximum expression levels in the cell type reached 30% of maximum expression levels across all cell types in that species (Extended Data Fig. 11a,b). We called a gene “absent”, when it was not reliably expressed in the cell type and its maximum expression levels in a cell type were below 30% of maximum expression levels across all cell types or the gene was not reliably expressed in the

cerebellum (not expressed in any cell type) in that species. Each orthologous gene group was then classified in each cell type according to the following rules: (I) a gene is considered to have conserved expression if it is "present" in all studied species; (II) a gene is considered as being expressed in a species-specific manner, if it is "present" in the focal species, "absent" in other species, and the pairwise fold-differences are more than five-fold; (III) a gene is classified as not expressed in a species-specific manner, if it is "absent" in the focal species, "present" in other species, and the pairwise fold-differences are more than five-fold; (IV) a gene is classified as not expressed (low) if it is "absent" in all studied species; (V) the remaining genes were assigned as "not classified".

In the comparative analyses of all three species (human, mouse, opossum), we polarized the presence/absence expression differences between mouse and human, using opossum as an outgroup species, and assigned the genes as having gained or lost expression in the mouse or human lineage. The differences between the eutherian and marsupial lineages cannot be polarized, because of the lack of a non-therian outgroup. Thus, if a gene is only expressed in opossum, we classified it as *marsupial-expressed*. Similarly, if a gene is not expressed specifically in opossum, we classified it as *eutherian-expressed*. In a comparison of human and mouse only, the genes with presence/absence expression differences were classified as *mouse-expressed* and *human-expressed*.

For each pair of species, we compared exonic length and GC content ratios among the genes within different presence/absence call classes, and used random forest models with exonic length and GC content as predictors to test if these could explain the presence/absence differences we called based on expression. The models were used with default parameters as implemented in scikit-learn (v1.1.1, python v3.9) RandomForestClassifier. As noted above, we detected some exonic length bias within the calls (Extended Data Fig. 11c). The length bias, however, could have both biological and technical sources, and random forest models were not able to predict the presence/absence expression differences between the species based on exonic length and GC content of the genes, indicating that these are not driving our calls (Extended Data Fig. 11d). We also evaluated the possible effects of using different 10x Chromium chemistry versions (v2 and v3) for data production on

classifying expression differences. For this we focussed on a subset of human and mouse datasets that have at least 2 biological replicates produced with Chromium v2 (E13.5, E14.5, E15.5, P7, P14 in mouse, and corresponding stages in human). We then replaced one of the human v2 replicates from each stage with a replicate produced with Chromium v3, and called presence/absence expression differences in the two subsets (mouse v2 : human v2, and mouse v2 : human v2/3) using the pipeline described above. Similar numbers of expression differences were detected using the two subsets (Extended Data Fig. 11f), indicating that our approach is robust to differences in Chromium versions.

Gene expression cell type-specificity.

To evaluate the cell type specificity of gene expression (Extended Data Fig. 11i), we determined specificity index τ , a metric described previously for evaluation of tissue-specificity[93]:

$$\tau = \frac{\sum_{i=1}^{n} 1 - \hat{x}_i}{n-1}$$

$$\hat{x}_i = \frac{x_i}{max(x)}$$,

where x is the normalized expression value for a certain gene across multiple cell types within a given species; x_i is the cell type i specific expression value; n is the total number of observed cell types. We determined the τ separately for each species using all cell types that were considered in the analysis of presence/absence expression differences (astroglia, oligodendrocytes, GABAergic deep nuclei neurons, Purkinje cells, interneurons, glutamatergic deep nuclei neurons, granule cells, UBCs). The index value 0 indicates broad expression, and 1 cell type-specific expression.

Gene ontology and pathway enrichment analyses.

For gene ontology and pathway enrichment analyses we used the WebGestaltR[94] package (version 0.4.4) and mouse functional databases of gene ontology and KEGG pathways as provided by WebGestalt (daily build accessed on 01.14.2019). The terms reported for principal components analyses (Fig. 3a and Extended Data

Fig. 8c) were identified as enriched (FDR<0.1) by gene set enrichment analyses based on gene loadings to principal components, and databases gene ontology *biological process noRedundant* and *KEGG*. Functional enrichments among the conserved markers (Extended Data Fig. 8f, Supplementary Table 9) were identified by over-representation analysis against the background of three-way 1:1 orthologues detected in all species (FDR < 0.05) using the functional databases *biological process noRedundant*, *molecular function noRedundant*, *cellular component noRedundant*, and *KEGG*. The molecular function terms, enriched among all conserved markers (Extended Data Fig. 8f), were manually grouped into broad categories. For each broad term category, we tested the enrichments among the conserved markers of individual cell states using hypergeometric tests and adjusted *P*-values using Benjamini-Hochberg method (Extended Data Fig. 8g). The gene lists for the respective terms were extracted from the WebGestalt[94] databases, the transcription factor list was downloaded from the animal TFDB (v.3.0)[61].

Functional enrichments among the genes with preserved trajectories assigned to different trajectory classes (Fig. 3f, Extended Data Fig. 9f) and among the genes with trajectory changes assigned to different lineages (Supplementary Table 12) were identified by over-representation analysis against the background of dynamic orthologous genes (FDR < 0.1). Functional enrichments among the genes with presence/absence expression differences in cerebellar cell types (Supplementary Table 12) were identified by over-representation analysis against the background of 7,047 orthologous genes included in the analysis (see *Presence/absence expression differences*; FDR < 0.1). Databases *biological process*, *molecular function*, *cellular component*, and *KEGG* were used.

Inference of transcription factor regulon activities

Transcription factor activities in mouse and human were estimated using the pySCENIC[36,95] pipeline. For computational purposes we subsampled the human dataset by keeping two (SN021 and SN105) out of the nine 8 wpc libraries (9581 out of 39 300 cells), resulting in a dataset of 151 237 cells. The whole mouse dataset was used. The cisTarget databases *500bpUp100Dw* (500 bp upstream and 100 bp downstream of the

transcription start site (TSS)) and *TSS+/-10kb* (10 kb upstream and downstream of the TSS) for mouse (mm10) and human (hg38), as well as the transcription factor to motif tables (version 9) were retrieved from the cisTarget website (https://resources.aertslab.org/ cistarget/). Gene regulatory networks were inferred using pySCENIC *grn* with the default settings. pySCENIC *ctx* was run with min_genes = 5, and *aucell* with the default settings. For each transcription factor, regulon activities (AUC scores) were z-scored across cell states.

Adult bulk RNA-sequencing data analyses.

The adult brain and cerebellum bulk RNA-sequencing data from nine mammals and chicken is from Brawand *et al*[47]. We used the reference genomes and orthology relationships from Ensembl release 91. Gene expression levels were measured as described by Wang *et al*.[60]. Briefly, for each gene we measured expression levels in the fragments per kilobase of coding DNA sequence (CDS) per million uniquely CDS-aligning reads (FPKM), a unit which corrects for both gene length and sequencing depth. We restricted the analysis to the coding regions of the longest protein-coding isoform of 1:1 orthologues that perfectly align across species.

Essential and disease-associated genes.

As a metrics of gene essentiality we used LOEUF scores (loss-of-function observed/expected upper bound fraction)[39] from the Genome Aggregation Database (gnomAD v2.1.1; https://gnomad.broadinstitute.org/downloads#v2-constraint), which ranks genes along a continuous spectrum of tolerance to loss-of-function variation based on human exome and genome sequencing data (*in vivo*). We obtained the human inherited disease gene list from the manually curated Human Gene Mutation Database (HGMD, PRO 17.1)[41], and only used genes with disease-causing mutations as described previously[40]. We used the genes that based on the mapping to the Unified Medical Language System (UMLS) were linked to the high level disease types 'Nervous system' or 'Psychiatric', and split these genes into two groups based on whether a gene was also linked to the high level disease type 'Developmental' (developmental n=373; other n=200). We obtained the cerebellum-linked disease gene lists from two sources. First, the curated lists of

genes associated with neurodevelopmental and adult-onset neurodegenerative disorders linked to cerebellar dysfunction are from Aldinger *et al.*[13]. Briefly, the cerebellar malformation list includes 54 genes combining published Dandy–Walker malformation and cerebellar hypoplasia genes and genes identified through exome sequencing; the Joubert syndrome list includes 42 published Joubert syndrome genes; the autism spectrum disorder list includes 108 high-confidence genes identified through exome and genome sequencing; the intellectual disability list includes 186 genes identified through exome sequencing; the spinocerebellar ataxia list includes 44 genes associated with OMIM phenotype PS164400. Second, the lists of driver genes associated with paediatric cancers is based on Gröbner *et al.*[42] and include 53 genes for medulloblastoma, 8 genes for ependymoma and 24 genes for pilocytic astrocytoma and pleomorphic xanthoastrocytoma.

Significance of categorical enrichments were evaluated using binomial tests using all dynamic genes as the background gene set (see *Identification of overdispersed genes* and *Gene expression trajectories*). Continuous differences were investigated using a permutation test (10,000 iterations) for $H_1 = smaller$ (LOEUF score). *P*-values were adjusted for multiple testing using Benjamini-Hochberg method.

General statistics and plots.

All statistical analyses and graphical representations were done in R (v.3.6.3 or v.4.1.2) using the R packages: tidyverse[96] v1.3.0; SingleCellExperiment[97] v1.6.0; liger[14] v0.4.6; rliger[98] v1.0.0; batchelor[65] v1.0.1; pheatmap[99] v1.0.12; ggplot2[100] v3.3.2.; ggExtra v0.10.0. Additionally, the following Python (v3.6) packages were used: scanpy[101] v1.5.1; htseq[102] v0.13.5.

SUPPLEMENTARY REFERENCES

56. Palkovits, M. Microdissection of Individual Brain Nuclei and Areas. in General Neurochemical Techniques (eds. Boulton, A. A. & Baker, G. B.) *Neuromethods* 1–17 (1985). doi:10.1385/0-89603-075-x:1.
57. Krishnaswami, S. et al. Using single nuclei for RNA-seq to capture the transcriptome of postmortem neurons. *Nature Protocols* **11**, 499–524 (2016).
58. Attar, M. et al. A practical solution for preserving single cells for RNA sequencing. *Scientific Reports* **8**, 2151 (2018).

59. Cunningham, F. et al. Ensembl 2019. *Nucleic Acids Res* **47**, gky1113- (2018).
60. Wang, Z.-Y. et al. Transcriptome and translatome co-evolution in mammals. *Nature* **588**, 642–647 (2020).
61. Hu, H. et al. AnimalTFDB 3.0: a comprehensive resource for annotation and prediction of animal transcription factors. *Nucleic Acids Res* **47**, gky822 (2018).
62. Scrucca, L., Fop, M., Murphy, T. B. & Raftery, A. E. mclust 5: Clustering, Classification and Density Estimation Using Gaussian Finite Mixture Models. *R J* **8**, 289–317 (2016).
63. Wolock, S. L., Lopez, R. & Klein, A. M. Scrublet: Computational Identification of Cell Doublets in Single-Cell Transcriptomic Data. *Cell Syst* **8**, 281-291.e9 (2019).
64. Melville, J. uwot: The Uniform Manifold Approximation and Projection (UMAP) Method for Dimensionality Reduction. *R package version 0.1.10.* https://CRAN.R-project.org/package=uwot (2020).
65. Haghverdi, L., Lun, A. T. L., Morgan, M. D. & Marioni, J. C. Batch effects in single-cell RNA-sequencing data are corrected by matching mutual nearest neighbors. *Nat Biotechnol* **36**, 421–427 (2018).
66. Zeisel, A. et al. Molecular Architecture of the Mouse Nervous System. *Cell* **174**, 999-1014.e22 (2018).
67. Visel, A., Thaller, C. & Eichele, G. GenePaint.org: an atlas of gene expression patterns in the mouse embryo. *Nucleic Acids Res* **32**, D552–D556 (2004).
68. Diez-Roux, G. et al. A High-Resolution Anatomical Atlas of the Transcriptome in the Mouse Embryo. *Plos Biol* **9**, e1000582 (2011).
69. Hashimoto, R. et al. Origins of oligodendrocytes in the cerebellum, whose development is controlled by the transcription factor, Sox9. *Mech Develop* **140**, 25–40 (2016).
70. Perlman, K. et al. Developmental trajectory of oligodendrocyte progenitor cells in the human brain revealed by single cell RNA sequencing. *Glia* (2020) doi:10.1002/glia.23777.
71. Hao, Y. et al. Integrated analysis of multimodal single-cell data. *Cell* 184, 3573-3587.e29 (2021).
72. Kharchenko, P., Petukhov, V. & Biederstedt, E. leidenAlg: Implements the Leiden Algorithm via an R Interface. *R package version 1.0.5*. https://github.com/kharchenkolab/leidenAlg (2021).
73. Team, S. D. RStan: the R interface to Stan. *R package version 2.21.2* http://mc-stan.org/ (2020).
74. Meredith, M. & Kruschke, J. HDInterval: Highest (Posterior) Density Intervals. *R package version 0.2.2*. https://CRAN.R-project.org/package=HDInterval (2020).
75. Phipson, B. et al. propeller: testing for differences in cell type proportions in single cell data. *Bioinformatics* **38**, 4720–4726 (2022).
76. Simmons, S. Cell Type Composition Analysis: Comparison of statistical methods. *bioRxiv* 2022.02.04.479123 (2022) doi:10.1101/2022.02.04.479123.
77. Büttner, M., Ostner, J., Müller, C. L., Theis, F. J. & Schubert, B. scCODA is a Bayesian model for compositional single-cell data analysis. *Nat Commun* **12**, 6876 (2021).
78. Giorgino, T. Computing and Visualizing Dynamic Time Warping Alignments in R : The dtw Package. *J Stat Softw* **31**, (2009).
79. Human Developmental Biology Resource (HDBR). HDBR Atlas. http://hdbratlas.org.
80. Kerwin, J. et al. The HUDSEN Atlas: a three-dimensional (3D) spatial framework for studying gene expression in the developing human brain. *J Anat* **217**, 289–299 (2010).
81. Gerrelli, D., Lisgo, S., Copp, A. J. & Lindsay, S. Enabling research with human embryonic and fetal tissue resources. *Development* **142**, 3073–3076 (2015).
82. Petukhov, V. et al. Cell segmentation in imaging-based spatial transcriptomics. *Nat Biotechnol* **40**, 345–354 (2022).
83. Biancalani, T. et al. Deep learning and alignment of spatially resolved single-cell transcriptomes with

Tangram. *Nat Methods* **18**, 1352–1362 (2021).
84. Saka, S. K. et al. Immuno-SABER enables highly multiplexed and amplified protein imaging in tissues. *Nat. Biotechnol.* **37**, 1080–1090 (2019).
85. Kishi, J. Y. et al. SABER amplifies FISH: enhanced multiplexed imaging of RNA and DNA in cells and tissues. *Nat Methods* **16**, 533–544 (2019).
86. Baglama, J., Reichel, L. & Lewis, B. W. irlba: Fast Truncated Singular Value Decomposition and Principal Components Analysis for Large Dense and Sparse Matrices. *R package version 2.3.3.* https://CRAN.R-project.org/package=irlba (2019).
87. Young, M. D. & Behjati, S. SoupX removes ambient RNA contamination from droplet-based single-cell RNA sequencing data. *Gigascience* **9**, giaa151 (2020).
88. Korsunsky, I. et al. Fast, sensitive and accurate integration of single-cell data with Harmony. *Nat Methods* **16**, 1289–1296 (2019).
89. Kumar, L. & Futschik, M. E. Mfuzz: A software package for soft clustering of microarray data. *Bioinformation* **2**, 5–7 (2007).
90. Brunson, J. C. & Read, Q. D. ggalluvial: Alluvial Plots in “ggplot2”. *R package version 0.12.3.* http://corybrunson.github.io/ggalluvial/ (2020).
91. Manno, G. L. et al. RNA velocity of single cells. *Nature* **560**, 494–498 (2018).
92. Kuo, A., Hansen, K. D. & Hicks, S. C. Quantification and statistical modeling of Chromium-based single-nucleus RNA-sequencing data. *bioRxiv* 2022.05.20.492835 (2022) doi:10.1101/2022.05.20.492835.
93. Yanai, I. et al. Genome-wide midrange transcription profiles reveal expression level relationships in human tissue specification. *Bioinformatics* **21**, 650–659 (2005).
94. Liao, Y., Wang, J., Jaehnig, E. J., Shi, Z. & Zhang, B. WebGestalt 2019: gene set analysis toolkit with revamped UIs and APIs. *Nucleic Acids Res* **47**, W199–W205 (2019).
95. Sande, B. V. de et al. A scalable SCENIC workflow for single-cell gene regulatory network analysis. *Nat Protoc* **15**, 2247–2276 (2020).
96. Wickham, H. et al. Welcome to the Tidyverse. *J Open Source Softw* **4**, 1686 (2019).
97. Amezquita, R. A. et al. Orchestrating single-cell analysis with Bioconductor. *Nat Methods* **17**, 137–145 (2020).
98. Liu, J. et al. Jointly defining cell types from multiple single-cell datasets using LIGER. *Nat Protoc* **15**, 3632–3662 (2020).
99. Kolde, R. pheatmap: Pretty Heatmaps. https://rdrr.io/cran/pheatmap/.
100. Wickham, H. ggplot2, Elegant Graphics for Data Analysis. (2009) doi:10.1007/978-0-387-98141-3.
101. Wolf, F. A., Angerer, P. & Theis, F. J. SCANPY: large-scale single-cell gene expression data analysis. *Genome Biol* **19**, 15 (2018).
102. Putri, G., Anders, S., Pyl, P., Pimanda, J. & Zanini, F. Analysing high-throughput sequencing data with HTSeq 2.0. https://htseq.readthedocs.io/en/master/ (2021).
103. Ábrahám, H., Tornóczky, T., Kosztolányi, G. & Seress, L. Cell formation in the cortical layers of the developing human cerebellum. *Int J Dev Neurosci* **19**, 53–62 (2001).
104. Yeung, J. et al. Wls Provides a New Compartmental View of the Rhombic Lip in Mouse Cerebellar Development. *J Neurosci* **34**, 12527–12537 (2014).
