# Supplementary material for: Cellular development and evolution of the mammalian cerebellum
Source: Nature. 2023 Nov 29;625(7996):788–96. doi: 10.1038/s41586-023-06884-x (PMC10808058; doi:10.1038/s41586-023-06884-x)
Supplement: Supplementary file 2 — Reporting Summary [file 41586_2023_6884_MOESM2_ESM.pdf]

nature portfolio

Corresponding author(s): Mari Sepp, Kevin Leiss, Ioannis Sarropoulos, Stefan M. Pfister, Henrik Kaessmann

Last updated by author(s): Nov 7, 2023

Reporting Summary

Nature Portfolio wishes to improve the reproducibility of the work that we publish. This form provides structure for consistency and transparency in reporting. For further information on Nature Portfolio policies, see our Editorial Policies and the Editorial Policy Checklist.

Statistics

For all statistical analyses, confirm that the following items are present in the figure legend, table legend, main text, or Methods section.

n/a	Confirmed	
☐	☒	The exact sample size (*n*) for each experimental group/condition, given as a discrete number and unit of measurement
☐	☒	A statement on whether measurements were taken from distinct samples or whether the same sample was measured repeatedly
☐	☒	The statistical test(s) used AND whether they are one- or two-sided<br>*Only common tests should be described solely by name; describe more complex techniques in the Methods section.*
☒	☐	A description of all covariates tested
☐	☒	A description of any assumptions or corrections, such as tests of normality and adjustment for multiple comparisons
☐	☒	A full description of the statistical parameters including central tendency (e.g. means) or other basic estimates (e.g. regression coefficient) AND variation (e.g. standard deviation) or associated estimates of uncertainty (e.g. confidence intervals)
☐	☒	For null hypothesis testing, the test statistic (e.g. *F*, *t*, *r*) with confidence intervals, effect sizes, degrees of freedom and *P* value noted<br>*Give P values as exact values whenever suitable.*
☐	☒	For Bayesian analysis, information on the choice of priors and Markov chain Monte Carlo settings
☒	☐	For hierarchical and complex designs, identification of the appropriate level for tests and full reporting of outcomes
☒	☐	Estimates of effect sizes (e.g. Cohen's *d*, Pearson's *r*), indicating how they were calculated

Our web collection on statistics for biologists contains articles on many of the points above.

Software and code

Policy information about availability of computer code

Data collection	BD FACSDiva 8.0.1 softaware was used for nuclei sorting.
Data analysis	Open source software including CellRanger v2 and v3; the R packages (v.3.6.3 and v.4.1.2) tidyverse v1.3.0, SingleCellExperiment v1.6.0, liger v0.4.6, rliger v1.0.0, batchelor v1.0.1, pheatmap80 v1.0.12, ggplot2 v3.3.2, ggExtra v0.10.0, mclust v5.4.3, uwot v0.1.10, Seurat v4.0.6, leidenAlg v1.0.5, HDInterval v0.2.2, dtw v1.20, irlba v2.3.3, SoupX, Harmony v1.0, Mfuzz v2.44.0, ggalluvial v0.12.3, WebGestaltR v0.4.4; the Python (v3.6) packages scanpy v1.5.1, htseq v0.13.5, Scrublet v0.2, Tangram v1.0.3, scikit-learn v1.1.1, pySCENIC; the Julia (v1.6.4) package Baysor v0.5.2; commercial software cellSens (Olympos), Fiji plugin Polylux (V1.9.0., Resolve Biosciences).<br>Custom code is available at https://gitlab.com/kaessmannlab/mammalian-cerebellum

For manuscripts utilizing custom algorithms or software that are central to the research but not yet described in published literature, software must be made available to editors and reviewers. We strongly encourage code deposition in a community repository (e.g. GitHub). See the Nature Portfolio guidelines for submitting code & software for further information.

Data

Policy information about availability of data

All manuscripts must include a data availability statement. This statement should provide the following information, where applicable:

- Accession codes, unique identifiers, or web links for publicly available datasets
- A description of any restrictions on data availability
- For clinical datasets or third party data, please ensure that the statement adheres to our policy

The datasets generated in the current study are available in the heiDATA repository, https://doi.org/10.11588/data/QDOC4E. Processed data can be interactively explored at https://apps.kaessmannlab.org/sc-cerebellum-transcriptome. Mouse and human processed data are also available as a CELLxGENE collection at https://

cellxgene.cziscience.com/collections/72d37bc9-76cc-442d-9131-da0e273862db. Previously published cerebellum snRNA-seq datasets are available at https://singlecell.broadinstitute.org/single_cell/study/SCP795 (Kozareva et al.), https://www.covid19cellatlas.org/aldinger20/ (Aldinger et al.), and https://github.com/linnarsson-lab/developing-human-brain (Braun et al.); and gnomAD LOEUF metrics (v2.1.1) at https://gnomad.broadinstitute.org/downloads#v2-constraint.

Field-specific reporting

Please select the one below that is the best fit for your research. If you are not sure, read the appropriate sections before making your selection.

☒ Life sciences ☐ Behavioural & social sciences ☐ Ecological, evolutionary & environmental sciences

For a reference copy of the document with all sections, see nature.com/documents/nr-reporting-summary-flat.pdf

Life sciences study design

All studies must disclose on these points even when the disclosure is negative.

Sample size	No statistical methods were used to predetermine sample size. At least 2 biological replicates were generated for each developmental stage (except human 20 wpc). All samples are listed in Supplementary Table 1, and an overview of the samples is given in Extended Data Fig. 1a. Human sample size was based on the number of individuals available, and comparable sample size was used for mouse and opossum.
Data exclusions	Low quality nuclei and mislabeled samples (not cerebellum) were excluded as described in the Methods.
Replication	At least 2 biological replicates were generated for each developmental stage (except human 20 wpc). Data from all replicates was included in the final dataset as long as the data quality and sample identity criteria were met (see Data exclusions).
Randomization	Randomization was not used in this study. Randomization was not relevant to our study since samples were not allocated into experimental groups.
Blinding	Blinding was not relevant to our study. Both data collection and analyses required an understanding of the nature of the sample being collected/analyzed.

Reporting for specific materials, systems and methods

We require information from authors about some types of materials, experimental systems and methods used in many studies. Here, indicate whether each material, system or method listed is relevant to your study. If you are not sure if a list item applies to your research, read the appropriate section before selecting a response.

Materials & experimental systems

n/a	Involved in the study	
☐	☒	Antibodies
☒	☐	Eukaryotic cell lines
☒	☐	Palaeontology and archaeology
☐	☒	Animals and other organisms
☐	☒	Human research participants
☒	☐	Clinical data
☒	☐	Dual use research of concern

Methods

n/a	Involved in the study	
☒	☐	ChIP-seq
☐	☒	Flow cytometry
☒	☐	MRI-based neuroimaging

Antibodies

Antibodies used	Lmx1a-Millipore:AB10533-lot:3868680; TBR2/EOMES-Millipore:ABN1687-Lot:Q3076145; TBR2/EOMES- Millipore:AB15894-lot:3090750; HCRTR2-R&D:MAB52461-lot:CBFY0115061; HCRTR2-Alomone Labs:AOR-002.<br><br>Donkey anti-Mouse IgG (H+L) Alexa Fluor 488 (Invitrogen, Cat.No.: A21202), Donkey anti-Mouse IgG (H+L) Alexa Fluor 568 (Invitrogen, Cat.No.: A10037), Donkey anti-Rabbit IgG (H+L) Alexa Fluor 488 (Invitrogen, Cat.No.: A21206), Donkey anti-Rabbit IgG (H+L) Alexa Fluor 568 (Invitrogen, Cat.No.: A10042), and Goat Anti-Chicken IgY (H+L) Alexa Fluor 568 (Abcam, Cat.No.:ab175477).
Validation	AB10533: evaluated by Western Blot in mouse testis tissue lysate by the provider; IHC signal in the rhombic lip shown by Yeung et al. 2014 (https://doi.org/10.1523/JNEUROSCI.1330-14.2014).<br>ABN1687: A representative lot detected TBR2 in Immunofluorescence applications (Nelson, B.R., et. al. (2013). J Neurosci. 33(21):9122-39; Hodge, R.D., et. al. (2013). J Neurosci. 33(9):4165-80), in immunohistochemistry applications (Yoon, K.J., et. al. (2008). Neuron. 58(4):519-31).<br>AB15894: a representative lot detected TBR2 in mouse cerebral cortex and mouse cerebellum tissue, as tested by the provider; staining in UBCs shown by Canton-Josh et al. 2022 (https://doi.org/10.7554/eLife.76912).<br>MAB52461: HCRTR2 was detected in immersion fixed paraffin-embedded sections of human brain (hypothalamus) by the provider.<br>AOR-002: IHC signal in the hypothalamus shown by Parekh et al. 2021 (https://doi.org/10.1038/s41598-021-00522-0).

Animals and other organisms

Policy information about studies involving animals; ARRIVE guidelines recommended for reporting animal research

Laboratory animals	RjOrl:SWISS and Bl6N mice (Mus musculus), gray short-tailed opossum (Monodelphis domestica). Animals from both sexes were used. Ages of the animals are listed in Supplementary Table 1, and an overview is given in Fig. 1a and Extended Data Fig. 1a. The animals were housed under a 12h/12h dark/light cycle (reversed for opossums) in a temperature (20-24 °C mouse; 24-26 °C opossum) and humidity (40-65% mouse, 60-65% opossum) controlled room with ad libitum access to food and water.
Wild animals	This study did not involve wild animals.
Field-collected samples	This study did not involve samples collected from the field.
Ethics oversight	All animal procedures were performed in compliance with national and international ethical guidelines for the care and use of laboratory animals, and were approved by the local animal welfare authorities: Heidelberg University Interfaculty Biomedical Research Facility (T-63/16, T-64/17, T-37/18, T-23/19), Vaud Cantonal Veterinary Office (No.2734.0) and Berlin State Office of Health and Social Affairs, LAGeSo (T0198/13, ZH104).

Note that full information on the approval of the study protocol must also be provided in the manuscript.

Human research participants

Policy information about studies involving human research participants

Population characteristics	Human samples were obtained from official scientific tissue banks. The samples come from healthy non-affected individuals defined as normal controls by the corresponding brain bank. Individuals from both sexes were included. For all samples, the sex and age are reported in Supplementary Table 1.
Recruitment	Informed consent for the use of tissues for research was obtained in writing from donors or their family.
Ethics oversight	The use of human samples was approved by an ERC Ethics Screening panel (associated with ERC Consolidator Grant 615253, OntoTransEvol) and ethics committees in Heidelberg (authorization S-220/2017), North East-Newcastle & North Tyneside (REC reference 18/NE/0290), London-Fulham (REC reference 18/LO/0822), Ministry of Health of Hungary (No.6008/8/2002/ETT) and Semmelweis University (No.32/1992/TUKEB).

Note that full information on the approval of the study protocol must also be provided in the manuscript.

Flow Cytometry

Plots

Confirm that:

☒ The axis labels state the marker and fluorochrome used (e.g. CD4-FITC).

☒ The axis scales are clearly visible. Include numbers along axes only for bottom left plot of group (a 'group' is an analysis of identical markers).

☐ All plots are contour plots with outliers or pseudocolor plots.

☒ A numerical value for number of cells or percentage (with statistics) is provided.

Methodology

Sample preparation	Nuclei were extracted from frozen tissues and stained with Hoechst
Instrument	BD FACSAria III
Software	BD FACSDiva 8.0.1
Cell population abundance	The nuclei were sorted based on the Hoechst staining. No quantifications were performed based on flow cytometry data. Further selection of barcodes containing a single nucleus was done based on RNA-sequencing data.
Gating strategy	To separate nuclei from the cellular debris the gates were set on FSC/SSC and at the excitation wavelength of 405 nm.

☒ Tick this box to confirm that a figure exemplifying the gating strategy is provided in the Supplementary Information.
